# Left Ventricular Ejection Fraction and Volumes: It Depends on the Imaging Method

**DOI:** 10.1111/echo.12331

**Published:** 2013-11-26

**Authors:** Peter W Wood, Jonathan B Choy, Navin C Nanda, Harald Becher

**Affiliations:** * Division of Cardiology, Mazankowski Alberta Heart InstituteEdmonton, Alberta, Canada; † Division of Cardiovascular Disease, University of Alabama at BirminghamBirmingham, Alabama

**Keywords:** echocardiography, contrast imaging, three-dimensional transthoracic echocardiography, left ventricular ejection fraction, left ventricular function

## Abstract

**Background and Methods:**

In order to provide guidance for using measurements of left ventricular (LV) volume and ejection fraction (LVEF) from different echocardiographic methods a PubMed review was performed on studies that reported reference values in normal populations for two-dimensional (2D ECHO) and three-dimensional (3D ECHO) echocardiography, nuclear imaging, cardiac computed tomography, and cardiac magnetic resonance imaging (CMR). In addition all studies (2 multicenter, 16 single center) were reviewed, which included at least 30 patients, and the results compared of noncontrast and contrast 2D ECHO, and 3D ECHO with those of CMR.

**Results:**

The lower limits for normal LVEF and the normal ranges for end-diastolic (EDV) and end-systolic (ESV) volumes were different in each method. Only minor differences in LVEF were found in studies comparing CMR and 2D contrast echocardiography or noncontrast 3D echocardiography. However, EDV and ESV measured with all echocardiographic methods were smaller and showed greater variability than those derived from CMR. Regarding agreement with CMR and reproducibility, all studies showed superiority of contrast 2D ECHO over noncontrast 2D ECHO and 3D ECHO over 2D ECHO. No final judgment can be made about the comparison between contrast 2D ECHO and noncontrast or contrast 3D ECHO.

**Conclusion:**

Contrast 2D ECHO and noncontrast 3D ECHO show good reproducibility and good agreement with CMR measurements of LVEF. The agreement of volumes is worse. Further studies are required to assess the clinical value of contrast 3D ECHO as noncontrast 3D ECHO is only reliable in patients with good acoustic windows.

Assessment of left ventricular (LV) function and volumes is the corner stone of cardiac diagnostics. Several imaging methods are in clinical practice and one would assume that these methods would provide the same results. However, if the same patient with stable conditions is investigated with different methods, different results are obtained which may have an impact on patient management. This review gives an overview on comparative studies between echocardiographic modalities and cardiac magnetic resonance imaging (CMR), which is regarded as the reference method for LV volumes and ejection fraction (LVEF).^1^ It will cover the normal values, comparative studies with CMR and a critical assessment of the reproducibility data. A recent article by Dorosz et al.^2^ has provided an extensive overview and a meta-analysis on studies comparing 2D and 3D echocardiography with CMR. Our review is complementary as it includes contrast echocardiography and provides a more comprehensive section on reproducibility, which has a major impact for clinical use.

## Methods

Normal values were selected from the guideline papers of the echocardiographic and radiological scientific societies or from articles which established the normal values. A pubmed review was carried out—including 18 studies, encompassing 1299 patients—comparing studies which included patients with abnormal LV function. Unlike the Dorosz article, the following review had an inclusion criteria of 30 patients or more per study and included contrast echocardiography and several more recent investigations in which Bland–Altman (BA) analysis was used as the method of calculating agreement with CMR in different patient groups. In these studies both patients with normal and abnormal hearts were included. In abnormal hearts the differences between the different imaging methods may become even more relevant.

BA analysis provides 2 parameters which allow to assess the agreement between different methods which measure the same parameter—bias and limits of agreement (LOA). Bias means the measurements with a specific echocardiographic technique are systematically different from the CMR measurements, which are regarded as the reference standard. A bias can be positive (= overestimation compared with the CMR measurements) or negative (= underestimation compared with the CMR measurements). For example, a bias of 5% means that the echocardiographic method overestimates the CMR measurements on an average by 5%. The LOA represent the degree of accuracy between the echocardiographic measurements and the CMR measurements. The LOA are calculated by 2 (or 1.96) standard deviations (SD) of the differences and covers the range of values which includes 95% of all the differences between the echocardiographic and CMR measurements. For example, with a bias of –5% and 2 SD = 10% the range is −15 to +5%. The smaller the LOA range the better is the agreement of the echocardiographic method when compared with CMR.^3^ In the review, we also included studies which used other method for assessment of the inter- and intra-observer variability such as intra-class correlation coefficients (ICC), and coefficient of variability (CV).

## Results

### Normal Values

The normal values for LV volumes and LVEF are shown in TablesI–III which have been extrapolated from different references (mean ± 2SD), with particular importance placed on the lower limits of LVEF, as these are clinical indicators for LV impairment. There are major discrepancies between modalities and processing techniques. There are also differences between gender and various ethnicities.^4^–^15^

**Table I:** Normal Values for Left Ventricular Ejection Fraction

Article	N	Mode	Male EF Lower Limit (%)	Female Lower Limit (%)
Alfakih et al.^4^	60	MRI TGE	57.0	58.0
Alfakih et al.^4^	60	MRI SSFP	55.0	54.0
Cain et al.^5^	96	MRI gradient echo*	49.0 (61–80 years)	53.0 (61–80 years)
Nikitin et al.^6^	95	MRI SSFP	66.0 (<65 years)	68.0 (>65 years)
			70.0 (>65 years)	72.0 (>65 years)
Lang et al. (ASE guidelines)^7^	510	2D ECHO	55.0	55.0
Aune et al.^8^	166	3D ECHO	49.0	49.0
Fukuda et al.^9^ – Japanese	410	3D ECHO (QLAB, TomTec)	51.0 (60–69 years)	53.0 (60–69 years)
Chahal et al.^10^ – European white	499	3D ECHO	50.0 (35–44 years)	52.0 (35–44 years)
			52.0 (45–54 years)	51.0 (45–54 years)
			48.0 (55–64 years)	53.0 (55–64 years)
			47.0 (65–75 years)	55.0 (65–75 years)
Chahal et al.^10^ – Indian Asian	479	3D ECHO	50.0 (35–44 years)	53.0 (35–44 years)
			51.0 (45–54 years)	52.0 (45–54 years)
			51.0 (55–64 years)	53.0 (55–64 years)
			53.0 (65–75 years)	55.0 (65–75 years)
Wang et al.^11^	140	gSPECT QGS	51.1	57.6
Wang et al.^11^	140	gSPECT 4D-MSPECT	57.1	51.5
Nakajima et al.^12^	268	gSPECT QGS	48.7	55.5
Hor et al.^13^	585	RNV	49.0	49.0
Pfisterer et al.^14^	1200	RNV	45.0	45.0
Jongjirasiri et al.^15^	115	320-CT	47.4	53.1

ASE = american society of echocardiography; MRI = magnetic resonance imaging; TGE = turbo gradient echo; SSFP = steady-state free procession; 2D = two-dimensional; 3D = three-dimensional; ECHO = echocardiography; gSPECT = gated single photon emission computed tomography; QGS = quantitative gated single photon emission computed tomography software; 4D-MSPECT = four-dimensional myocardial single photon emission computed tomography; RNV = radionuclide ventriculography; 320-CT = 320 slice computed tomography.

* Sequence not specified.

**Table II:** Normal Values for Left Ventricular End-Diastolic Volume Index from the Literature

Article	N	Mode	Male EDV Lower Limit (mL)	Male EDV Upper Limit (mL)	Female EDV Lower Limit (mL)	Female EDV Upper Limit (mL)
Alfakih et al.^4^	60	MRI TGE	45.0 (40–65 years)	104.0 (40–65 years)	48.0 (40–65 years)	94.0 (40–65 years)
Alfakih et al.^4^	60	MRI SSFP	53.0 (40–65 years)	112.0 (40–65 years)	56.0 (40–65 years)	99.0 (40–65 years)
Cain et al.^5^	96	MRI gradient echo*	48.0 (51–60 years)	97.0 (51–60 years)	46.0 (51–60 years)	87.0 (51–60 years)
			43.0 (61–70 years)	92.0 (61–70 years)	45.0 (61–70 years)	86.0 (61–70 years)
			36.0 (71–80 years)	88.0 (71–80 years)	44.0 (71–80 years)	87.0 (71–80 years)
Nikitin et al.^6^	95	MRI SSFP	63.0 (<65 years)	73.0 (<65 years)	63.0 (<65 years)	73.0 (<65 years)
			54.0 (>65 years)	67.0 (>65 years)	56.0 (>65 years)	69.0 (>65 years)
Lang et al. (ASE guidelines)^7^	510	2D ECHO	35.0	75.0	35.0	75.0
Aune et al.^8^	166	3D ECHO	46.0	86.0	42.0	74.0
Fukuda et al.^9^ – Japanese	410	3D ECHO (QLAB, TomTec)	21.0 (50–59 years)	69.0 (50–59 years)	28.0 (50–59 years)	60.0 (50–59 years)
			20.0 (60–69 years)	68.0 (60–69 years)	25.0 (60–69 years)	57.0 (60–69 years)
Chahal et al.^10^ – European White	499	3D ECHO	N/A	72.0 (35–44 years)		64.0 (35–44 years)
				71.0 (45–54 years)		59.0 (45–54 years)
				64.0 (55–64 years)		56.0 (55–64 years)
				62.0 (65–75 years)		52.0 (65–75 years)
Chahal et al.^10^ – Indian Asian	479	3D ECHO	N/A	63.0 (35–44 years)	N/A	59.0 (35–44 years)
				57.0 (45–54 years)		53.0 (45–54 years)
				55.0 (55–64 years)		49.0 (55–64 years)
				56.0 (65–75 years)		60.0 (65–75 years)
Wang et al.^11^	140	gSPECT QGS	17.6	62.4	14.7	51.1
Wang et al.^11^	140	gSPECT 4D-MSPECT	15.4	60.2	12.8	53.2
Nakajima et al.^12^	268	gSPECT QGS	27.5	74.1	17.9	60.7
Hor et al.^13^*	585	RNV	130.0	160.0	130.0	160.0
Jongjirasiri et al.^15^*	115	320-CT	88.0	157.2	61.7	128.1

Values are indexed to body surface area. ASE = american society of echocardiography; MRI = magnetic resonance imaging; TGE = turbo gradient ECHO; SSFP = steady-state free procession; 2D = two-dimensional; 3D = three-dimensional; ECHO = echocardiography; gSPECT = gated single photon emission computed tomography; QGS = quantitative gated single photon emission computed tomography software; 4D-MSPECT = four-dimensional myocardial single photon emission computed tomography; RNV = radionuclide ventriculography; 320-CT = 320 slice computed tomography.

* Values were not indexed.

† Sequence not specified.

**Table III:** Normal Values for Left Ventricular End-Systolic Volume Index from the Literature

Article	N	Mode	Male ESV Lower Limit (mL)	Male ESV Upper Limit (mL)	Female ESV Lower Limit (mL)	Female ESV Upper Limit (mL)
Alfakih et al.^4^*	60	MRI TGE	19.7 (40–65 years)	78.9 (40–65 years)	22.0 (40–65 years)	56.0 (40–65 years)
Alfakih et al.^4^*	60	MRI SSFP	26.1 (40–65 years)	89.7 (40–65 years)	26.8 (40–65 years)	68.8 (40–65 years)
Cain et al.^5^	96	MRI gradient echo†	14.0 (51–60 years)	46.0 (51–60 years)	13.0 (51–60 years)	37.0 (51–60 years)
			12.0 (61–70 years)	44.0 (61–70 years)	14.0 (61–70 years)	38.0 (61–70 years)
			8.0 (71–80 years)	43.0 (71–80 years)	14.0 (71–80 years)	39.0 (71–80 years)
Nikitin et al.^6^	95	MRI SSFP	19.0 (<65 years)	24.0 (<65 years)	19.0 (<65 years)	23.0 (<65 years)
			15.0 (>65 years)	20.0 (>65 years)	13.0 (>65 years)	20.0 (>65 years)
Lang et al. (ASE guidelines)^7^	510	2D ECHO	12.0	30.0	12.0	30.0
Aune et al.^8^	166	3D ECHO	17.0	41.0	13.0	33.0
Fukuda et al.^9^ – Japanese	410	3D ECHO	7.0 (50–59 years)	27.0 (50–59 years)	8.0 (50–59 years)	24.0 (50–59 years)
			7.0 (60–69 years)	27.0 (60–69 years)	7.0 (60–69 years)	23.0 (60–69 years)
Chahal et al.^10^ – European White	499	3D ECHO	30.0 (35–44 years)	N/A	26.0 (35–44 years)	N/A
			32.0 (45–54 years)		26.0 (45–54 years)	
			29.0 (55–64 years)		21.0 (55–64 years)	
			26.0 (65–75 years)		20.0 (65–75 years)	
Chahal et al.^10^ – Indian Asian	479	3D ECHO	28.0 (35–44 years)	N/A	23.0 (35–44 years)	N/A
			24.0 (45–54 years)		21.0 (45–54 years)	
			23.0 (55–64 years)		19.0 (55–64 years)	
			24.0 (65–75 years)		22.0 (65–75 years)	
Wang et al.^11^	140	gSPECT QGS		26.6		17.3
Wang et al.^11^	140	gSPECT 4D-MSPECT		20.4		20.1
Nakajima et al.^12^	268	gSPECT QGS		33.2		23.7
Hor et al.^13^*	585	RNV	50.0	60.0	50.0	60.0
Jongjirasiri et al.^15^*	115	320-CT	28.4	68.0	15.9	52.3

Values are indexed to body surface area. ASE = american society of echocardiography; MRI = magnetic resonance imaging; TGE = turbo gradient ECHO; SSFP = steady-state free procession; 2D = two-dimensional; 3D = three-dimensional; ECHO = echocardiography; gSPECT = gated single photon emission computed tomography; QGS = quantitative gated single photon emission computed tomography software; 4D-MSPECT = four-dimensional myocardial single photon emission computed tomography; RNV = radionuclide ventriculography; 320-CT = 320 slice computed tomography.

* Values were not indexed.

† Sequence not specified.

### Studies Comparing Echocardiographic Methods with CMR

**Figure 1:**
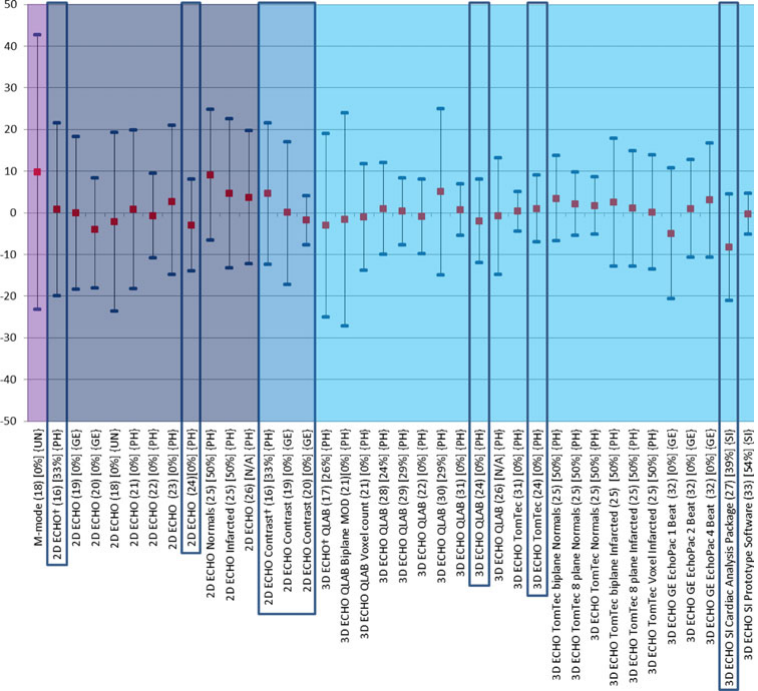
Comparison of echocardiographic techniques with cardiac magnetic resonance imaging for measurement of ejection fraction (%). Red square box indicates bias compared with magnetic resonance imaging. Blue line at each end of the plots indicates the lower and upper limits of agreement calculated by Bland–Altman. 2D ECHO = two-dimensional echocardiography; 3D ECHO = three-dimensional echocardiography; NSR = normal sinus rhythm; MOD = method of disks; QLAB = Philips online and offline LV volume calculation tool; TomTec = offline left ventricular volume calculation tool. † indicates multicenter studies. Values in square brackets are the percentage of patients without disease within each study.

**Figure 2:**
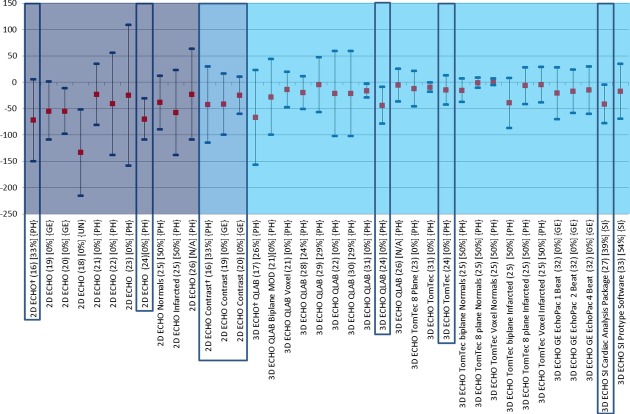
Comparison of echocardiographic techniques with cardiac magnetic resonance imaging for measurement of end-diastolic volume (mL). Red square box indicates bias compared with magnetic resonance imaging. Blue line at each end of the plots indicates the lower and upper limits of agreement calculated by Bland–Altman. MRI = magnetic resonance imaging; 2D ECHO = two-dimensional echocardiography; 3D ECHO = three-dimensional echocardiography; NSR = normal sinus rhythm; MOD = method of disks; QLAB = Philips online and offline LV volume calculation tool; TomTec = offline left ventricular volume calculation tool.

**Figure 3:**
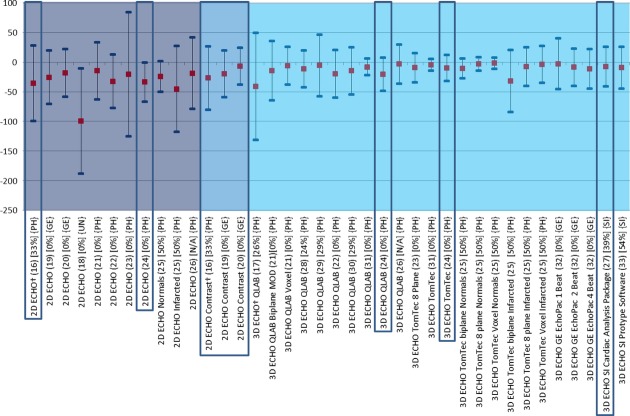
Comparison of echocardiographic techniques with cardiac magnetic resonance imaging for measurement of end-systolic volume (mL). Red square box indicates bias compared with magnetic resonance imaging. Blue line at each end of the plots indicates the lower and upper limits of agreement calculated by Bland–Altman. MRI = magnetic resonance imaging; 2D ECHO = two-dimensional echocardiography; 3D ECHO = three-dimensional echocardiography; NSR = normal sinus rhythm; MOD = method of disks; QLAB = Philips online and offline LV volume calculation tool; TomTec = offline left ventricular volume calculation tool.

Only 2 multicenter studies have been performed to compare CMR with echocardiographic imaging modalities (marked by a † in Figs.1–3). Hoffman et al. investigated 120 patients with variable levels of LV function, of which 55 patients had CMR as well as standard and contrast two-dimensional echocardiography (2D ECHO). They showed in BA analysis for unenhanced 2D ECHO (Simpson's biplane) LVEF to have a good agreement (bias = 0.8%; LOA = −20.0% to 21.6%) with CMR. Contrast-enhanced 2D ECHO (Simpson's biplane) showed a similar agreement (bias = 4.6%; LOAs of −12.4% to 21.6%). End-diastolic volume (EDV) and end-systolic volume (ESV) in unenhanced 2D ECHO showed a bias of −72.3 mL (LOA = −150.3 to 5.7 mL) and −35.7 mL (LOA = −99.4 to 28 mL), respectively, compared with −42.3 mL (LOA = −114.6 to 30 mL) and −27.2 mL (LOA = −80.9 to 26.5 mL) using contrast 2D ECHO. Various combinations of 3 readers (1 onsite and 2 offsite) produced mean percentage errors (MPE) and confidence intervals (95% CI) for 2D ECHO (12.8, 10.9–14.8; 11.7, 10.1–13.4; 12.6, 10.4–14.8) and for contrast 2D ECHO (8.9, 7.5–10.3; 8.8, 7.5–10.2; 4.1, 3.1–5.0). These showed a clear improved agreement when contrast echocardiographic agents were used.^16^ The second multicenter study, consisting of 92 patients with various degrees of LV function as assessed by Simpson's biplane LVEF assessment, was carried out by Mor-Avi et al. investigating the accuracy and reproducibility of three-dimensional echocardiography (3D ECHO) (5 beat volume acquisition; QLAB, Philips Ultrasound Ltd., Bothell, Washington, USA). The bias (LOAs) were −3% (LOA = ±22%), −67 mL (LOA = ±90 mL), and −41 mL (LOA = ±90 mL) for LVEF, EDV, and ESV, respectively. The degree of bias in the volume calculations was attributed to the less experienced centers in 3D ECHO utilization, which highlights the importance of adequate training in the utilization of 3D ECHO for LV function assessment.^17^

There are 16 single center studies which satisfy the inclusion criteria of this review; 3 of which included ≥100 patients (highlighted by a box in Figs.1–3). Whereas native and contrast echocardiography can be performed with all state of the art scanners, there are currently only 4 commercially available systems for 3D ECHO with their specific analysis software (Philips Ultrasound Ltd., Bothell, WA, USA, GE Healthcare, Salt Lake City, UT, USA, Siemens Healthcare, Erlangen, Germany, and Toshiba America Medical Systems Inc., California, USA). In addition, there is one commercially available software for off line analysis (TomTec Imaging Systems, Untershleissheim, Germany). No studies have been performed to compare measurements obtained on scanners from different manufacturers. The single center studies included a total of 1087 patients and healthy volunteers. The bias and LOA for measurements of LVEF, EDV, and ESV are shown in Figures3.^16^–^33^ The findings in single and multicenter studies can be concluded as follows:

1. 2D contrast echocardiography is superior to 2D noncontrast echocardiography regarding agreement of volume and LVEF measurements. The volumes measured with 2D contrast echocardiography are closer to the corresponding CMR measurements than those obtained with noncontrast echocardiography. Contrast 2D echocardiography is particularly useful in patients with poor acoustic windows.^16^,^19^,^20^
2. Most studies showed the superiority of noncontrast 3D over noncontrast 2D ECHO.^16^–^33^ In particular the measured volumes deviated less from the CMR measurements using noncontrast 3D ECHO compared with noncontrast 2D ECHO. Only one study specifically focused on patients with LV aneurysms and seemed to show similar results compared with the other studies.^22^
3. There are several studies exploring different recording and analysis protocols for noncontrast 3D ECHO. The most frequently used technique for 3D ECHO volume measurements is voxel count. The borders of the LV cavity are traced semiautomatically and the voxels (known volume) inside the traced volume are counted. The difference between the analysis software from different manufacturers is the number of 2D slices, which are used for the initial tracing of the endocardium. Whereas, QLAB uses 2 orthogonal views, TomTec uses at least 3 planes, however, after segmentation all further measurements are performed via voxel count. Jacobs et al.^21^ showed better results using the 3D voxel counting method compared with biplane Simpson obtained from a 3D dataset. Voxel count was also superior to multiplane measurements of LVEF.^23^,^25^ There was no significant difference in LVEF estimation between the QLAB and TomTec voxel methods.^29^,^31^ However, the TomTec volume measurements were closer to CMR than the QLAB measurements.^24^
4. Most 3D studies used a multibeat acquisition: that means that the 3D dataset is acquired by small datasets, which are acquired during 4 or more consecutive beats and are electronically stitched together. A study by Macron et al. investigated the impact of single beat acquisition (which is associated with limited temporal and spatial resolution) versus multibeat 3D ECHO image acquisition. The single beat acquisition resulted in significantly smaller and more variable measurements of ejection fraction (bias 5%) compared with 4 beat acquisitions.^32^ Thavendiranathan et al.^33^ used a real time scanner (Siemens, CA, USA), which provides high volume rates and showed good agreement with CMR. They also were able to scan patients with atrial fibrillation. The authors went on to report the effect of adding various amounts of adjustments to the endocardial border of the contour algorithm, demonstrating a closer relationship with CMR in LVEF measurements when the contour finding algorithm is moved slightly outside the initially traced contour so as to include the small LV trabeculations.
5. No final judgment can be made about the comparison between 2D contrast and 3D noncontrast and contrast studies. No study yet fulfilled the inclusion criteria for this review, but there is a European multicenter study completed, which will be available within a year. Caiani et al. compared 3D ECHO with 2D ECHO (Simpson's biplane) and CMR in a population of 46 patients of which a subset of 14 consented for contrast infusion during 3D ECHO acquisition. The LVEF was not different with both methods, but the agreement of EDV and ESV became worse when a contrast agent was used, the bias (LOA) for contrast EDV was −34 mL compared to −5.7 mL for native 3D ECHO. It was suggested by the authors that this negative impact of values relative to the reference method may have been due to bubble destruction, resulting from the high density of scanlines required for full volumetric acquisition.^26^ In a recent study of Thavendiranathan and colleagues the reproducibility of noncontrast 3D ECHO exceeded that of 2D and 3D contrast echocardiography. But, this study included only patients with good image quality and no CMR measurements were performed.^34^

### Observer Variability in the Comparative Studies

Different methods of statistical analysis were used to assess the reproducibility of tests between 2 different observers and of repeat tests for the same observer; ICC, BA method, CV, and percentage difference of the mean (MD).^3^,^35^ Pearson's correlation coefficient was also used in one article. Generally, using ICC as the statistical test for assessing reproducibility, 3D ECHO was more reproducible than 2D ECHO. With BA analysis there was an obvious difference between the 2 methods, although, BA was not used very often for comparison in 3D ECHO. The most frequently used test for 3D ECHO was MD, defined as the absolute difference between corresponding repeated measurements expressed in percentage of their mean, which showed an improvement of 3D ECHO as compared with 2D ECHO (TablesIV–VI).

**Table IV:** Two-Dimensional Echocardiography, Inter-Observer and Intra-Observer Comparison

Technique	Study Reference	Statistic	Inter-Observer			Intra-Observer		
			LVEF	EDV	ESV	LVEF	EDV	ESV
Simpson's biplane	Malm et al.^20^*	BA	±15.4%	±25.7 mL	±20 mL	±9.45%	N/A	N/A
Simpson's biplane	Jacobs et al.^21^	MD	14 ± 17	19 ± 20	24 ± 21	13 ± 11	13 ± 21	24 ± 24
		BA	±18%	±42 mL	±20 mL	±12%	±46 mL	±24 mL
Simpson's biplane	Caiani et al.^26^	CV	14.2†	26.4†	37.7†			
		ICC				N/A	0.91	0.92
Simpson's biplane	Gutierrez-Chico et al.^23^	ICC	0.94	0.58	0.83	0.92	0.80	0.89
Simpson's biplane	Hoffman et al.^16^	ICC	0.79	N/A	N/A	N/A	N/A	N/A
Simpsons biplane contrast	Hoffman et al.^16^	ICC	0.91	N/A	N/A	N/A	N/A	N/A
Simpsons biplane contrast	Malm et al.^20^*	BA	±6.4%	±20.7 mL	±15.2 mL	±3.95%	N/A	N/A
3D ECHO biplane (TomTec)	Gutierrez-Chico et al.^23^	ICC	0.96	0.97	0.99	0.97	0.98	0.97

ICC = intra-class correlation coefficient; CV = coefficient of variability (%); BA = Bland–Altman (limits of agreement ± 2SD); MD = mean difference expressed as a percentage of the mean (% ± 2SD); EDV = end-diastolic volume; ESV = end-systolic volume; LVEF = ejection fraction; CI = confidence interval.

* Bias not made available.

† Standard deviation not reported.

**Table V:** Three-Dimensional Echocardiography Inter-Observer and Intra-Observer Comparison of the Literature with Varying Methods of Statistical Analysis

Technique	Study Reference	Statistic	Inter-Observer			Intra-Observer		
			LVEF	EDV	ESV	LVEF	EDV	ESV
5 Bt (QLAB)	Mor-Avi et al.^17^	CV	N/A	8 ± 16	13 ± 28	N/A	5 ± 10	10 ± 22
4 Bt (QLAB)	Jacobs et al.^21^	MD	5 ± 4	10 ± 8	11 ± 6	10 ± 5	10 ± 6	11 ± 5
		BA	3 ± 4%	14 ± 20 mL	7 ± 10 mL	6 ± 6%	13 ± 14 mL	6 ± 6 mL
4 Bt (TomTec)	Sugeng et al.^29^	MD	10.5 ± 16.6	11.2 ± 17.2	14.2 ± 23.6	5.6 ± 6.8	3.9 ± 4	5.6 ± 7.8
4 Bt (QLAB)	Soliman et al.^31^	MD	9.7 ± 8.8	12.2 ± 10.1	13.6 ± 11.2	7.3 ± 9.1	7.2 ± 8.1	9.1 ± 7.2
4 Bt (TomTec)	Soliman et al.^31^	MD	7.1 ± 6.9	6.4 ± 7.8	7.8 ± 9.7	6.6 ± 7.4	4.7 ± 3.2	6.1 ± 5.8
1 Bt (EchoPAC)	Macron et al.^32^	MD	8.6 ± 23.2	9.2 ± 11.2	11.9 ± 16.8	6.8 ± 8.8	3.4 ± 7.4	8.0 ± 10.2
2 Bt (EchoPAC)	Macron et al.^32^	MD	6.6 ± 7.8	4.6 ± 8.4	9.0 ± 13.8	4.5 ± 7.8	3.2 ± 6.6	3.2 ± 4.8
4 Bt (EchoPAC)	Macron et al.^32^	MD	9.2 ± 9.6	5.6 ± 7.2	9.6 ± 14.8	6.4 ± 12.8	3.1 ± 5.4	4.2 ± 10.6
1 Bt, Online	Chang et al.^27^	ICC		0.99	0.99		0.99	0.99
		BA	N/A	−1.62 ± 8.78 mL	−0.32 ± 10.0 mL	N/A	−7.91 ± 33.06 mL	−1.62 ± 6.85 mL
4 Pl (TomTec)	Gutierrez-Chico et al.^23^	ICC	0.98	0.99	0.99	0.99	0.99	0.99
8 Pl (TomTec)	Gutierrez-Chico et al.^23^	ICC	0.99	0.99	0.99	0.99	0.99	0.99
4 Bt (TomTec)	Qi et al.^30^	PCC	0.98	0.995	0.998	0.948	0.947	0.982
3 to 5 Bt (Siemens)	Thavendiranathan et al.^33^	MD	1 ± 16	9 ± 14	9 ± 16	2 ± 20	5 ± 20	3 ± 22

PCC = Pearson's correlation coefficient; Bt = beat; Pl = plane; ICC = intra-class correlation; CV = coefficient of variability (% ± 2SD); BA = Bland–Altman (Bias ± 2SD); MD = mean difference expressed as a percentage of the mean (% ± 2SD); EDV = end-diastolic volume; ESV = end-systolic volume; LVEF = ejection fraction.

**Table VI:** Cardiac Magnetic Resonance Imaging, Inter-Observer and Intra-Observer Comparison, Obtained from Studies in which Cardiac Magnetic Resonance Imaging and Echocardiographic Methods are Compared

Technique	Study Reference	Statistic	Inter-Observer			Intra-Observer		
			LVEF	EDV	ESV	LVEF	EDV	ESV
CMR	Hoffman et al.^16^	ICC	0.86; 95% CI 0.80–0.92	N/A	N/A	N/A	N/A	N/A
CMR	Mor-Avi et al.^17^	MD	N/A	5 ± 8	7 ± 14	N/A	4 ± 10	4 ± 8
CMR	Sugeng et al.^29^	MD	8.5 ± 19.4	6.3 ± 11.4	7.7 ± 13.2	6.2 ± 12.4	2.4 ± 4.6	6.3 ± 9.2
CMR	van Geuns et al.^50^	MD	5.6 ± 6.0	3.7 ± 3.1	4.8 ± 4.0	0.2 ± 6.2	0.2 ± 1.0	1.4 ± 2.3
CMR	Thavendiranathan et al.^33^	MD	1 ± 4	1 ± 12	2 ± 10	1 ± 4	0 ± 8	0 ± 12

CMR = cardiac magnetic resonance imaging; ICC = intra-class correlation; MD = mean difference expressed as a percentage of the mean (% ± 2SD); EDV = end-diastolic volume; ESV = end-systolic volume; LVEF = ejection fraction; CI = confidence interval.

The reproducibility of CMR measurements is better than that measured with noncontrast 2D ECHO in most studies. But with contrast echocardiography there are only minor differences in particular for LVEF—in the multicenter study of Hoffman et al. contrast 2D ECHO had a better variability using ICC than did CMR (0.91 vs. 0.86, respectively) when the onsite reader and 2 offsite readers were compared.^16^ The inter- and intra-observer variability of CMR measurements are dependent on the expertise of the readers.^36^ The inter-observer variability of LVEF measurements can be improved from 7.2% to 3.7% after training.^36^ In TableVI the studies are listed in which the reproducibility was reported for CMR. The variability of computed tomography (CT) and radionuclide ventriculography (RNV) reported by separate studies are listed in TableVII; all of which reported excellent variability except one study which reported a correlation coefficient of 0.6 for LVEF by RNV. This is not surprising as CT utilizes the same methods for border delineation and volume calculation as CMR using images with higher spatial resolution.^37^

**Table 7:** Computed Tomography and Radionuclide Ventriculography, Inter-Observer and Intra-Observer Comparison

Technique	Study Reference	Statistic	Inter-Observer			Intra-Observer		
			LVEF	EDV	ESV	LVEF	EDV	ESV
CT Multirow	Raman et al.^51^	ICC	0.98	0.98	0.99	N/A	N/A	N/A
CT 64-Slice	Annuar et al.^52^	ICC	0.99 ± 0.01	N/A	N/A	N/A	N/A	N/A
CT 64-Slice	Maffei et al.^37^	CV	4.4	2.3	3.8	1.3	1.0	1.3
CT 64-Slice	Sarwar et al.^53^	PCC	0.75	0.91	0.87	N/A	N/A	N/A
RNV	Xie et al.^54^	PCC	0.98	0.98	0.98	0.99	0.99	0.99
RNV	Sibille et al.^55^	CV	0.6	1.1	1.7	N/A	N/A	N/A

CT = computed tomography; RNV = radionuclide ventriculography; ICC = intra-class correlation; CV = coefficient of variability (% ± 2SD); EDV = end-diastolic volume; ESV = end-systolic volume; LVEF = ejection fraction. PCC = Pearson's correaltion coeffcient.

## Discussion

This is the most comprehensive review on echocardiographic methods for measurement of ejection fraction. We have indeed included the entire spectrum of available echocardiographic methods for assessment of LV function. A recent review and meta-analysis of Dorosz et al.^2^ provided an excellent summary of native 2D and 3D ECHO, but did not include other frequently used technologies such as contrast echocardiography. There are only 2 more recent articles comparing 2D and 3D ECHO with CMR (Chang et al. and Thavendiranathan et al.^27^,^33^). That warrants no new meta-analysis on this topic. For the comparison of contrast echocardiography or M mode echocardiography with CMR there were not enough studies to justify a meta-analysis.

### Normal Values

It is important to have a reference point from which to compare values. In a perfect world one normal range should apply for all cardiac imaging tools in calculating ejection fraction and volumetric measurements. However, it is becoming apparent that due to the differences in methodology and algorithms between diagnostic modalities a fixed value is not possible, and so it is necessary to develop a range of normal values corresponding to specific modalities. This may even be the case for various software packages used in the same diagnostic tool (TablesI–III). The reference values are based on studies involving cohorts as low as 60 patients ranging up to 1200.

In circumstances such as monitoring of treatment with potentially cardiotoxic drugs (trastuzumab), accurate assessment of LVEF is crucial. However, if measurements are used interchangeably between different tests, which may be occurring in current practice, then interpretation may become difficult and the information could be misleading. A normal ejection fraction for CMR may correspond with a mildly compromised ventricle in 2D ECHO, and so on. Thus, the difference between these measurements may be the difference between whether a patient does, or does not, qualify for therapeutic intervention. The differences in normal values are particularly large when EDV and ESV are compared (TablesII and III). It should be acknowledged that if techniques are used interchangeably an improvement or deterioration may be observed on an individual basis which does not reflect the patient's underlying pathology.

### Comparison of Echocardiographic Studies with CMR

CMR has been regarded the reference standard for measurement of LV volumes and ejection fraction because of its high image quality and volumetric data.^1^ There are well-performed ex vivo studies which have demonstrated the validity of CMR measurements.^38^ The bias and 95% LOA between the dog heart model data and different methods for LV volume determination were between 4.94 ± 12.11 mL and 1.71 ± 18.11mL. High image quality with good segmentation of blood and tissue as well as a volumetric dataset are the prerequisites for accurate measurements of LV volumes.

We have not included several older techniques which were used prior to the introduction of Simpson's biplane method in 2D ECHO, such as M-mode and linear methods of LV functional assessment, including Quinones et al., Dumesnil et al., Baran et al., and Teicholz et al.^39^–^42^ The early methods for assessment of LVEF such as the Quinones method involved diameter measurements on 2D echocardiograms to calculate LVEF based on a mathematical calculation assuming an ellipsoid shape. While innovative at the time, the Quinones method could not adapt to other LV morphologies other than ellipsoid shapes. These methods are not recommended any more by the American and European Societies of Echocardiography.^7^,^43^ This is due to proven inaccuracies in ventricles with abnormal shapes and regional wall-motion abnormalities. To our knowledge there are no studies comparing these methods with CMR.

One of the difficulties facing LV functional assessment is that LVEF may be a moving target as a beat to beat variability has been reported up to 5.8 ± 1.7%. LVEF varies with BP, inotropic state and heart rate. To obtain reliable comparison of LVEF measurements from 2 different methods it is mandatory to examine the patient under the same hemodynamic conditions. The effect of the beat to beat variation can only be minimized by taking multiple measurements and averaging the results.^44^,^45^ However, in reality this is often not carried out due to time constraints and high clinical loads.

There is no systematic difference in the measured ejection fraction between the echocardiographic methods and CMR (Fig.1). As already reported for the normal values there are major differences in volumes between echocardiographic methods and CMR. With the use of contrast agents these differences in volume measurement have reduced, however, this is still not to the level where results can be considered interchangeable. Regarding volumes 3D ECHO has been demonstrated to show a large improvement toward the values of CMR, and as such, is the most accurate ultrasound technique for determining LV function, although the total number of patients included in trials is still small and very good acoustic windows are needed.^46^ The most promising technique in echocardiography is certainly contrast 3D, however, the excellent results demonstrated by the Jenkins group in 2009 could not be reproduced by Caiani et al., and so further investigation is required.^47^ TableVIII summarizes the advantages and limitations of the different echocardiographic methods.

**Table 8:** Advantages and Limitations of Echocardiographic Techniques Used for Ventricular Functional Assessment

Method	Assessment Type	Geometrical Assumption	Advantages	Limitations
Linear	M-mode	Yes	Quick and easy to perform	Assumes an ellipsoid shaped ventricle Needs perpendicular parasternal imaging Depends on acoustic window Therefore, least accurate method
2D	Simpson's biplane	Yes	More accurate and reproducible than M-mode.	Assumes an ellipsoid shaped ventricle Needs unforeshortened orthogonal views Depends on acoustic window and operator experience Endocardium often not fully visualized in a single frame used for manual tracing
2D contrast	Simpson's biplane	Yes	More accurate and reproducible than 2D Less susceptible to poor image quality	As 2D; but less susceptible to poor image quality
3D biplane	Simpson's biplane	Yes	2 orthogonal planes from the same beat Avoids off-axis views and foreshortening	Assumes an ellipsoid shaped ventricle Depends on acoustic window and operator experience Full volume recordings require stable heart rhythm and breath hold (usually 4 beats) otherwise stitching artifacts Real time acquisition reduces image quality Lower spatial and temporal resolution than 2D
3D	Voxel count	Partial	Avoids off-axis views and foreshortening Automatic border delineation following minimal landmark allocations More accurate than 2D and 3D biplane	Depends on acoustic window and operator experience Full volume recordings require stable heart rhythm and breath hold (usually 4 beats) otherwise stitching artifacts Real time acquisition reduces image quality Lower spatial and temporal resolution than 2D Has problems fitting to some abnormal LV shapes (i.e. apical infarcts)
3D contrast	Voxel count	Partial	Best agreement with CMR and CT angiography	Few studies available Artifacts from apical contrast destruction and attenuation Lowest spatial and temporal resolution Not all software packages can perform LV assessment with the addition of contrast

LV = left ventricle; 2D = two-dimensional; 3D = three-dimensional; CMR = cardiac magnetic resonance imaging; CT = computed tomography.

The reproducibility of the echocardiography techniques showed a marked improvement with the introduction of contrast 2D ECHO and 3D ECHO in both intra-observer and inter-observer methods and comes close to CMR. However, we think there is not yet enough data to provide benchmarks for quality assessment. The differing tests used for variability assessments make it difficult for a reliable assessment to be made—in particular as some studies do not include either intra- or inter-observer calculations. Considering the importance of accurate assessment of LV function it is remarkable that there has been only a limited body of comprehensive studies which allow to define the differences between the different imaging methods. In particular, the data on the reproducibility are not satisfactory. The scientific societies should encourage studies or registries to broaden the database and to provide guidelines on how to perform validation studies. The BA analysis appears to be an ideal test to analyze differences between methods or between observers. Based on the available studies the different imaging techniques for assessment of LVEF and volumes are not interchangeable. If follow-up measurements are necessary they should be performed with the same method.

In this review, only 2 of the 18 studies reviewed as validation studies for echocardiography were multicenter studies. Most of the data are from single center studies, which are subject to referral bias. Thus, the reproducibility may be overestimated. Further investigation from larger cohorts is needed.

### Is Visual Assessment an Alternative?

Visual assessment of LV function on 2D echocardiograms has been used in many hospitals; for example, by estimating the LVEF in 5% steps such as 30–35% or just classifying the LV function as normal, mildly, moderately, or severely impaired. The reason for using a visual rather than a quantitative assessment is the extra time needed to calculate LV volumes and difficulties to trace the endocardial borders on still frames. To our knowledge no studies using visual assessment have been published in contrast echocardiography, where endocardial borders usually are well seen. Although visual assessment of global LV function has been reported to be “reasonable” among experienced readers, the actual inter-observer variability was 5.8%.^48^ This does not allow the use of visual assessment for follow-up studies of LVEF and volumes. In CMR a comparison of visual and quantitative assessment of LVEF showed a major underestimation (8.4%) with visual assessment. Therefore, it was recommended to use quantitative analysis for accurate assessment of LV function.^49^
